# Core–Shell Structured Silica-Coated Iron Nanowires Composites for Enhanced Electromagnetic Wave Absorption Properties

**DOI:** 10.3390/ijms24108620

**Published:** 2023-05-11

**Authors:** Pingan Yang, Wenxian Ye, Haibo Ruan, Rui Li, Mengjie Shou, Yichen Yin, Xin Huang, Yuxin Zhang, Jiufei Luo

**Affiliations:** 1 School of Automation, Chongqing University of Posts and Telecommunications, Chongqing 400065, China; yangpa@cqupt.edu.cn (P.Y.); lirui@cqupt.edu.cn (R.L.); shoumj@cqupt.edu.cn (M.S.); huangxin@cqupt.edu.cn (X.H.); 2 Chongqing Key Laboratory of Materials Surface & Interface Science, Chongqing University of Arts and Sciences, Chongqing 402160, China; 3 College of Material Science and Engineering, Chongqing University, Chongqing 400044, China; zhangyuxin@cqu.edu.cn

**Keywords:** core–shell structure, Fe NWs@SiO_2_, microwave absorption

## Abstract

In this study, we successfully prepared core–shell heterostructured nanocomposites (Fe NWs@SiO_2_), with ferromagnetic nanowires (Fe NWs) as the core and silica (SiO_2_) as the shell. The composites exhibited enhanced electromagnetic wave absorption and oxidation resistance and were synthesized using a simple liquid-phase hydrolysis reaction. We tested and analyzed the microwave absorption properties of Fe NWs@SiO_2_ composites with varied filling rates (mass fractions of 10 wt%, 30 wt%, and 50 wt% after mixing with paraffin). The results showed that the sample filled with 50 wt% had the best comprehensive performance. At the matching thickness of 7.25 mm, the minimum reflection loss (*RL_min_*) could reach −54.88 dB at 13.52 GHz and the effective absorption bandwidth (EAB, *RL <* −10 dB) could reach 2.88 GHz in the range of 8.96–17.12 GHz. Enhanced microwave absorption performance of the core–shell structured Fe NWs@SiO_2_ composites could be attributed to the magnetic loss of the composite, the core–shell heterogeneous interface polarization effect, and the small-scale effect induced by the one-dimensional structure. Theoretically, this research provided Fe NWs@SiO_2_ composites with highly absorbent and antioxidant core–shell structures for future practical applications.

## 1. Introduction

With the rapid development of modern electronic information and communication technology in many fields, a large number of electromagnetic waves with different energies and rich spectrum have been produced [1,2,3]. However, excessive electromagnetic waves can cause serious electromagnetic radiation pollution problems, which in turn endanger human health [4,5,6]. To solve the growing problem of electromagnetic radiation pollution, it is urgent to develop electromagnetic wave absorbing materials (EMWAs) with excellent absorbing properties, which can convert electromagnetic waves into heat or other forms of energy [7,8,9].

Among many EMWAs, the ferromagnetic absorbing materials [10,11,12] (Fe, Co, Ni, and their alloys) with high permeability, high magnetic loss, high Snoek’s cut-off frequency, high saturation magnetization, and high Curie temperature have shown excellent electromagnetic wave loss ability [13,14,15]. However, ferromagnetic absorbing materials have the disadvantages of high density and strong skin effects [16]. Therefore, many researchers have studied ferromagnetic absorbing materials with different microstructures to overcome the above shortcomings, such as three-dimensional Fe nanohollow spherical structures [17,18,19], two-dimensional Fe nanosheet structures [20,21,22], and one-dimensional Fe nanowire structures [23,24,25]. Among them, the one-dimensional Fe nanowire (NW) structure has the advantages of small size, large specific surface area, and the ability to improve the anisotropy and resonance frequency of magnetic materials [24] that exhibit good microwave absorption properties. For example, Shen et al. [23] reported necklace-like Fe NWs with a high aspect ratio, with the the minimum reflection loss (*RL_min_*) reaching −27.28 dB at 3.68 GHz. Yang et al. [26] also reported large-sized Fe NWs; the results showed that the *RL_min_* of −44.67 dB at 2.72 GHz and the effective absorption bandwidth (EAB) reached 8.56 GHz at a layer thickness of 1.42 mm. This kind of one-dimensional nanowire structure exhibited excellent microwave absorption performance, which provided a reference for high-performance magnetic metal absorbing materials [27,28].

The single component Fe NWs with high dielectric constant, poor impedance matching characteristics [29], and poor chemical stability can hinder their practical application [30]. To obtain EMWAs with excellent performance, researchers have proposed a strategy of combining ferromagnetic materials with dielectric materials to construct core–shell composite materials [31,32]. By coating Fe NWs with a layer of dielectric material to form a core–shell structure, the dielectric shell will effectively reduce the dielectric constant of Fe NWs, optimize the impedance matching, improve the magnetic loss and dielectric loss, and thus enhance the absorption efficiency. For example, Wang et al. [33] reported that the dielectric carbon shells were coated on the surface of Fe nanofibers, which improved the oxidation resistance and reduced the density, optimized the impedance matching, and obtained a better microwave absorption performance. Yang et al. [34] also reported that dielectric silver shells were coated on the surface of Fe NWs; the results confirmed that the dielectric silver shells could reduce the dielectric constant, optimize the impedance matching, and enhance the microwave absorption performance. As an excellent dielectric material, silica (SiO_2_) can not only adjust the dielectric loss capacity of composites but can also improve the corrosion resistance and wear resistance of composites [35,36]. After coating SiO_2_ on the surface of magnetic metal, the permeability will not be suppressed and the electromagnetic wave (EMW) can more easily be absorbed into the material internally rather than by reflection [37]. Hence, SiO_2_ can often be applied to improve the microwave absorption properties of ferromagnetic materials. For example, Pao et al. [38] reported that SiO_2_ nanoparticles coated Co nanosheets, confirming that SiO_2_ nanoparticles can regulate the dielectric constant, increase the dielectric loss, optimize the impedance matching, and improve the microwave absorption performance. Therefore, SiO_2_ shell-coated Fe NWs would be a good choice and an effective way to reduce dielectric constant, optimize impedance matching, and improve microwave absorption performance and oxidation resistance.

In this work, core–shell heterogeneous nanocomposites with Fe NWs as core and SiO_2_ as shell Fe NWs@SiO_2_ were successfully obtained by a simple liquid-phase hydrolysis method. This core–shell structure enriched the heterogeneous interface, reduced the dielectric constant, optimized the impedance matching, and resulted in improved microwave absorption performance and chemical stability. Here, electromagnetic parameters and permeability of the Fe NWs@SiO_2_ composites were obtained by the coaxial testing method and the wave-absorbing properties of Fe NWs@SiO_2_ composites with mass filling fractions of 10 wt%, 30 wt%, and 50 wt% were calculated and analyzed. This work provided guiding significance for the development of high-efficiency magnetic metal-based microwave absorption materials.

## 2. Results and Discussion

**Figure 1:**
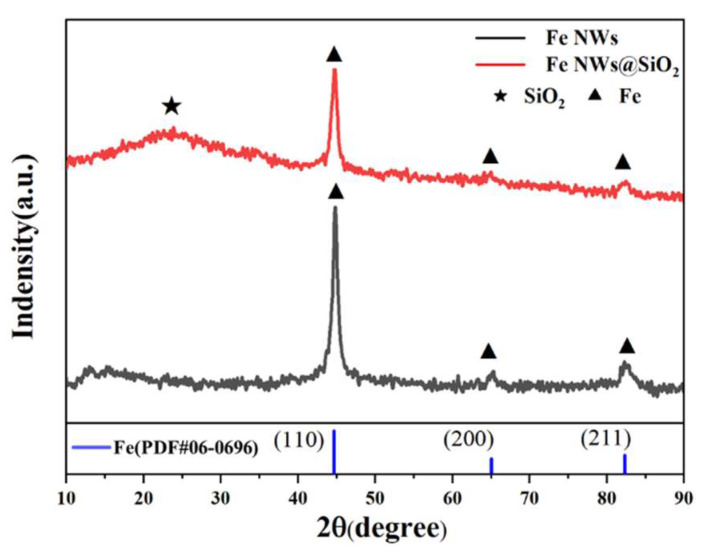
XRD spectra of Fe NWs and Fe NWs@SiO_2_ composites.

The XRD patterns of Fe NWs and Fe NWs@SiO_2_ are shown in Figure 1. Diffraction peaks of Fe NWs were about 2θ = 44.7, 65.0, and 82.4°, corresponding to (110), (200), and (211) planes of body-centered cubic (bcc) α-Fe (JCPDS No. 06-0696), respectively [24]. Diffraction peaks of Fe NWs@SiO_2_ composite were 2θ = 44.7, 65.0, and 82.4°, corresponding to (110), (200), and (211) crystal planes of the body-centered cube, respectively. In addition, a wide diffraction peak at 20–30° appeared in the Fe NWs@SiO_2_ spectrum, which was not found in the Fe NWs’ spectrum, indicating that the surface of Fe NWs was covered with SiO_2_. Therefore, it was proved that SiO_2_ was successfully coated on the surface of Fe NWs. To further determine the chemical composition of Fe NWs@SiO_2_ composites, other means of research will be continued.

**Figure 2:**
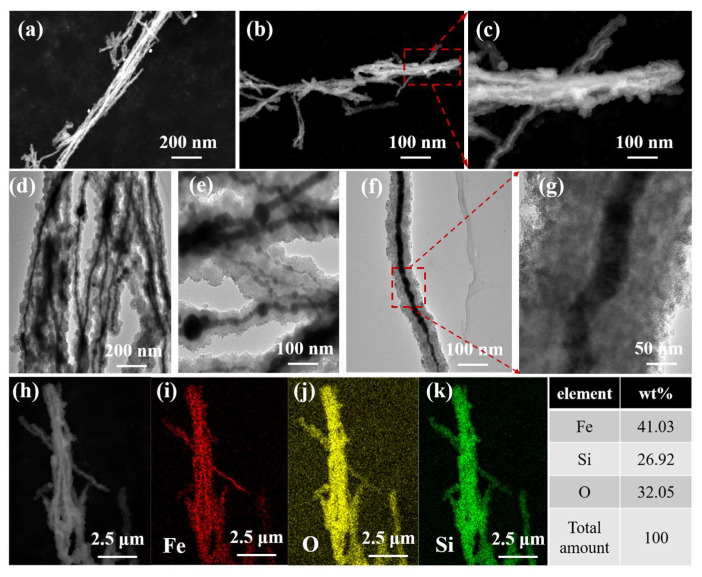
The SEM images of (**a**–**c**), TEM images of (**d**–**g**), and (**h**–**k**) EDS mapping mode of Fe NWs@SiO_2_ composites.

**Figure A1:**
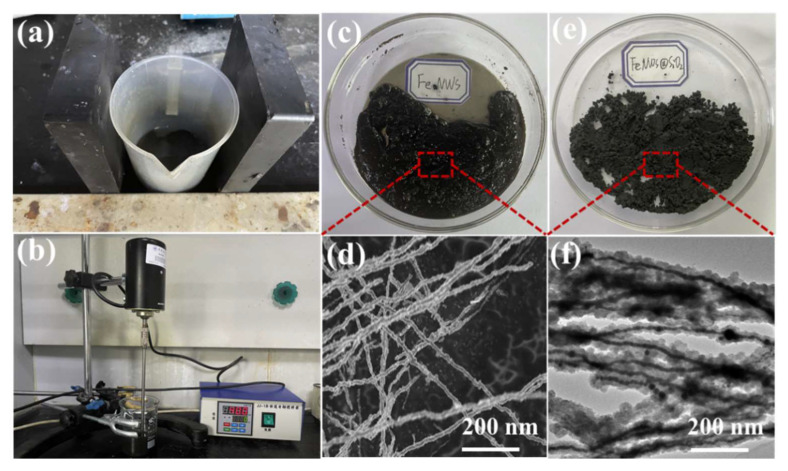
(**a**) Preparation of Fe NWs by a magnetic field, (**b**) preparation of Fe NWs@SiO_2_ by mechanical agitation, (**c**) real photos of the Fe NWs, (**d**) real photos of the Fe NWs@SiO_2_, (**e**) SEM image of Fe NWs, and (**f**) TEM image of core–shell Fe NWs@SiO_2_.

The morphology, elemental composition, and microstructure of Fe NWs@SiO_2_ composites were characterized by SEM and TEM, as shown in Figure 2. The composite material can be seen in Figure 2a as a nanowire structure with an average diameter of 100 nm. Figure 2b,c shows the microscopic morphology of Fe NWs@SiO_2_ at the same position with different magnifications; the Fe NWs were surrounded by nanoparticles and exhibited an obvious core–shell structure. To further determine the microstructure of the composites, TEM will be used for characterization. It can be seen from Figure 2d,e that the Fe NWs were tightly wrapped; this phenomenon can be more clearly seen from the enlarged images of Figure 2f,g. Moreover, the wrapping effect became uniform and dense and the core–shell structure appeared very complete and homogeneous (a comparison between pure Fe NWs and coated Fe NWs is shown in Figure A1d,f). DES was used to analyze the composition of the elements in the above core–shell structure and the mapping images of each element are shown in Figure 2h–k. Element scanning was performed in the area of Figure 2h; Fe, O, and Si elements were detected in the core–shell structure. The distribution of each element was linear, with Fe elements concentrated in the core and O and Si elements uniformly and densely overlaid with Fe in a linear distribution. From the microstructure and elemental analysis, it could be determined that Fe NWs were coated by SiO_2_ and formed a core–shell structure.

**Figure 3:**
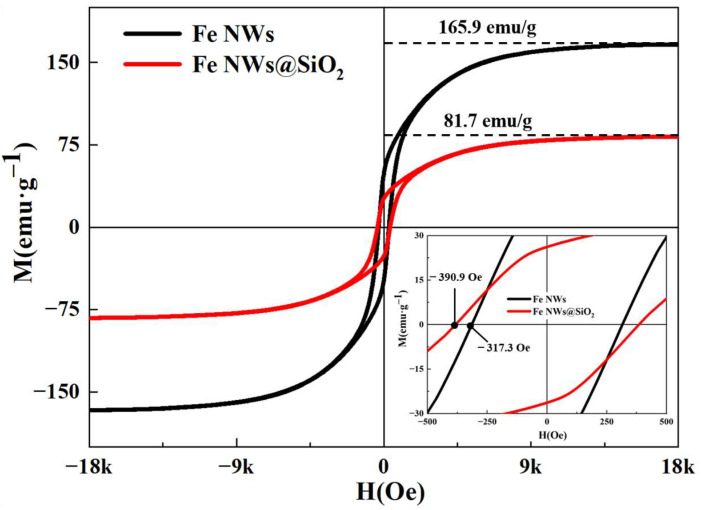
Hysteresis lines of Fe NWs and Fe NWs@SiO_2_ composites.

To further determine whether SiO_2_ on the surface of Fe NWs affected magnetic properties, hysteresis loops of Fe NWs and Fe NWs@SiO_2_ (*M*-*H* curve) were measured at 300 K, from which saturation magnetization (*M_s_*) degree and coercivity (*H_c_*) were obtained, as shown in Figure 3. It can be seen that both particles exhibited typical ferromagnetic properties, with the magnetization intensity of the particles varying with the increase in the applied magnetic field and then reaching saturation [39]. The *M_s_* values of nanocomposites are known to be related to the magnetic composition and are proportional to the Fe content [37]. However, SiO_2_, as an antimagnetic material, does not contribute to the saturation magnetization, but the SiO_2_ shell layer can reduce the magnetic moment per unit volume of Fe NWs@SiO_2_, which has a great impact on the magnetic properties of Fe NWs@SiO_2_. Therefore, the increase of SiO_2_ shell content will reduce the *M_s_* value of Fe NWs. It can be seen that the *M_s_* (81.7 emu/g) value of Fe NWs@SiO_2_ was higher than that of Fe NWs (165.9 emu/g) [40]. When the magnetocrystalline anisotropy energy, stress, and impurity content of the material increased, its *H_c_* also increased [41]. As shown in Figure 3, the *H_c_* value of Fe NWs@SiO_2_ (390.9 Oe) was higher than that of Fe NWs (317.3 Oe). The SiO_2_ shell led to magnetocrystalline anisotropy and interfacial stress on the surface of Fe NWs@SiO_2_, resulting in a significant increase in *H_c_*. High *H_c_* values will contribute to shifting the resonant frequency to a higher region [42]. Thus, the higher *H_c_* value of Fe NWs@SiO_2_ composites improved the microwave absorption performance in the higher frequency range.

According to the transmission line theory [43], the reflection loss value (*RL*) can be calculated according to the electromagnetic parameters of the material ($\varepsilon_{r},\;\mu_{r})$; the specific formula is as follows [44]:

(1) $$RL\left(\mathrm{dB}\right)=20\mathrm{lg}\left|\frac{Z_{in}-Z_{0}}{Z_{in}+Z_{0}}\right|$$

(2) $$Z_{in}=Z_{0}\sqrt{\frac{\mu_{r}}{\varepsilon_{r}}}\tanh(j\frac{2\pi fd}{c}\sqrt{\mu_{r}\varepsilon_{r}})$$

where $Z_{in}$ is the intrinsic impedance of the material; $Z_{0}$ is the intrinsic impedance of air; $\varepsilon_{r}$ and $\mu_{r}$ are the complex dielectric constant and complex permeability of the material, respectively; f is the electromagnetic wave frequency; d is the corresponding thickness of the material; c is the speed of light in a vacuum.

**Figure 4:**
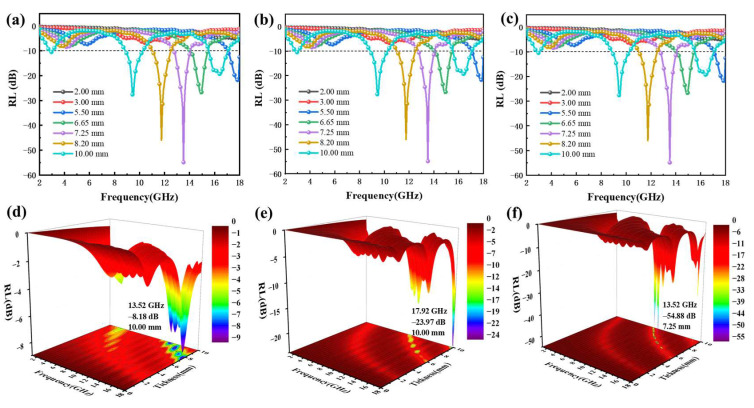
One-dimensional (1D) and 3D plots of reflection loss values with frequency and thickness for Fe NWs@SiO_2_ composites at different filling ratios: 10 wt% (**a**,**d**), 30 wt% (**b**,**e**), and 50 wt% (**c**,**f**).

**Figure A3:**
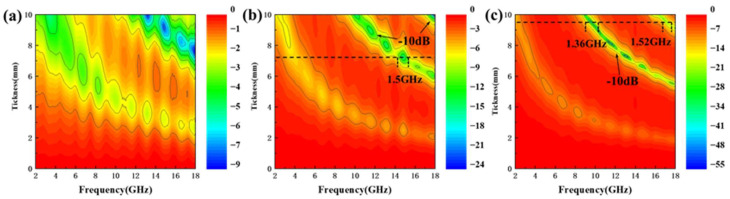
The 2D plots of reflection loss values with frequency and thickness for Fe NWs@SiO_2_ composites at different filling ratios: 10 wt% (**a**), 30 wt% (**b**), and 50 wt% (**c**).

According to the above theory, the microwave absorption characteristics of Fe NWs@SiO_2_ composite materials with different filling mass fractions were studied. Figure 4 shows the simulated calculated reflection curves in the frequency range of 2–18 GHz for the composites prepared by mixing 10 wt%, 30 wt%, and 50 wt% of Fe NWs@SiO_2_ with paraffin, respectively. The figures in the first and the second row represent one-dimensional (1D) and three-dimensional (3D) plots of the *RL* values of the three filling mass fractions as a function of frequency and thickness, respectively. In the range of 2–18 GHz, when the filling mass fraction was 10 wt%, the *RL* value could not reach −10 dB, even if the thickness was adjusted (Figure 4a,d). Figure A3 shows that the EAB of 10 wt%, 30 wt%, and 50 wt% Fe NWs@SiO_2_ composites were 0 GHz, 1.5 GHz, and 2.88 GHz, respectively. In addition, as shown in Figure 4e,f, when the matching thickness was 10 mm and 7.25 mm, the *RL_min_* of the sample (30 wt% and 50 wt%) reached −23.97 dB and −54.88 dB, respectively. Overall, the comprehensive absorption performance was better at a filling mass fraction of 50 wt%.

**Figure 5:**
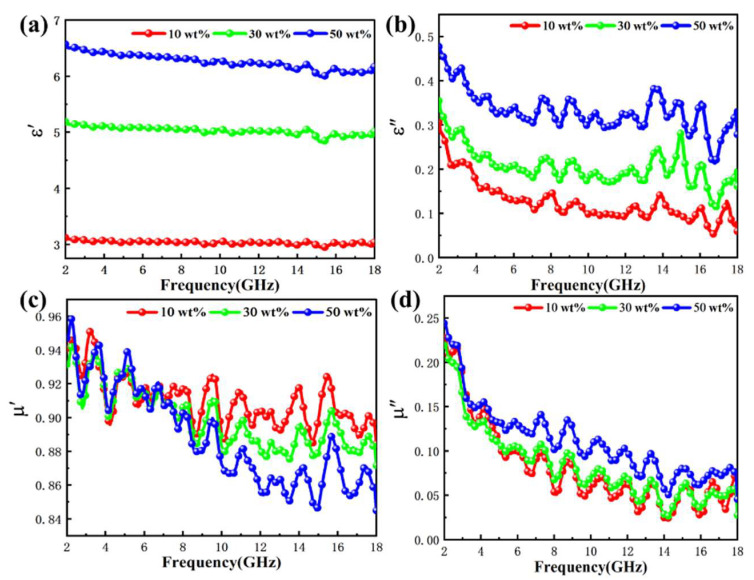
Frequency dependence of (**a**) real part and (**b**) imaginary part of complex permittivity and (**c**) real part and (**d**) imaginary part of the relative complex permeability of the Fe NWs@SiO_2_ composites with different filling ratios.

Electromagnetic parameters are an important basis for evaluating the absorption capacity; the complex permittivity ($\varepsilon_{r}=\varepsilon^{\prime }-j\varepsilon^{\prime \prime }$) and complex permeability ($\mu_{r}=\mu^{\prime }-j\mu^{\prime \prime }$) of these parameters depend on the frequency of electromagnetic waves [45]. The real part of the complex permittivity and complex permeability characterizes the ability of a material to store electrical energy and magnetic energy, while the imaginary part characterizes the ability of a material to dissipate electrical energy and magnetic energy, respectively [46]. To further investigate the microwave absorption mechanism of Fe NWs@SiO_2_, the complex permittivity and permeability of Fe NWs@SiO_2_ were analyzed. The $\varepsilon^{\prime }$ and $\varepsilon^{\prime \prime }$ curves of Fe NWs@SiO_2_ with different filling mass fractions are shown in Figure 5a,b. The curves of *ε*′ values for Fe NWs@SiO_2_ absorbing materials with different filling ratios showed similar trends in the frequency test range, with a smooth decreasing trend. Since SiO_2_ has good electrical insulation properties, the SiO_2_ coating formed an insulating layer on the surface of Fe NWs, thus hindering the electron displacement polarization of Fe NWs and reducing the $\varepsilon^{\prime }$ value. In addition, when Fe NWs@SiO_2_ was added to paraffin to form composites, the electronic shifts and interfacial polarization of Fe NWs intrinsically strengthened as the filling ratio of Fe NWs@SiO_2_ increased; thus, the $\varepsilon^{\prime }$ value increased. As shown in Figure 5a, the value of $\varepsilon^{\prime }$ increased with the increase in the filling ratio. The SiO_2_ shell layer also reduced the conductivity of the mixed Fe NWs, which in turn weakened the conductivity loss capacity of the mixed Fe NWs, resulting in a consequent decrease in their $\varepsilon^{\prime \prime }$. The $\varepsilon^{\prime \prime }$ value curves of the three filling ratios had similar decreasing trends in the frequency test range, with more disorderly changes and many rebound peaks, which may have originated from dielectric loss mechanisms such as dipole polarization and interfacial polarization. In this paper, the reported changes in the $\varepsilon^{\prime }$ and $\varepsilon^{\prime \prime }$ curves may have been a synergistic effect of space charge, interface, and orientation polarization induced by the one-dimensional core–shell structure of Fe NWs@SiO_2_ [47].

**Figure 6:**
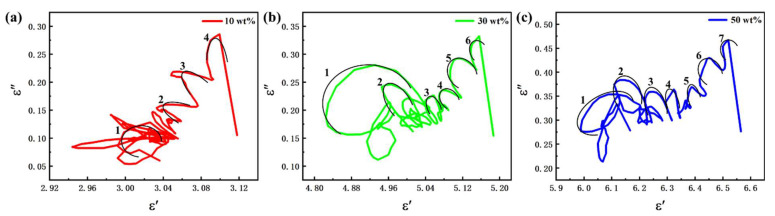
$\varepsilon^{\prime }$-$\varepsilon^{\prime \prime }$ plot of Fe NWs@SiO_2_-paraffin sample with 10 wt% loading (**a**), 30 wt% loading (**b**), and 50 wt% loading (**c**).

The dielectric loss of Fe NWs@SiO_2_ composites was further analyzed. Dielectric loss includes conduction loss, ion polarization, electron polarization, interfacial polarization, and dipole relaxation [48]. Among them, ion polarization and electron polarization were not considered in the study frequency range. Therefore, the effects of conduction loss, interfacial polarization, and dipole relaxation on dielectric loss were mainly studied. According to this theory of Debye, each Debye relaxation in the $\varepsilon^{\prime }$-$\varepsilon^{\prime \prime }$ graph corresponded to a Cole–Cole semicircle, which could be expressed by the following formula [49]:

(3) $${\left(\varepsilon^{\prime }-\frac{\varepsilon_{S}+\varepsilon_{\infty }}{2}\right)}^{2}+{\left(\varepsilon^{\prime \prime }\right)}^{2}={\left(\frac{\varepsilon_{S}-\varepsilon_{\infty }}{2}\right)}^{2}$$

where $\varepsilon_{S}$ and $\varepsilon_{\infty }$ are the static permittivity and relative permittivity of the high-frequency limit, respectively. The $\varepsilon^{\prime }$-$\varepsilon^{\prime \prime }$ plots for different filling mass fractions of Fe NWs@SiO_2_ composites are shown in Figure 6a–c. As can be seen in the figure, the $\varepsilon^{\prime }$-$\varepsilon^{\prime \prime }$ plots with filling ratios of 10 wt%, 30 wt%, and 50 wt% showed 4, 6, and 7 strong and distorted semicircles, respectively, which were caused by multiple relaxation processes occurring inside the material for electromagnetic waves. In addition, the one-dimensional core–shell structure of Fe NWs@SiO_2_ led to an asymmetric distribution of space charges, which generated a large number of dipoles and dipole polarization, as well as interfacial polarization at the two interfaces of the core–shell. The appearance of a straight “tail” at the end of the $\varepsilon^{\prime }$-$\varepsilon^{\prime \prime }$ curve with different filling ratios indicated the coexistence of multiple loss mechanisms [50]. Therefore, various Debye relaxation and other dielectric loss mechanisms existed in the core–shell composites of Fe NWs@SiO_2_.

The $\mu^{\prime }$ and $\mu^{\prime \prime }$ curves for different Fe NWs@SiO_2_ filling mass fractions are shown in Figure 5c,d. The main dissipation mechanism of magnetic materials is represented by $\mu^{\prime }$ and $\mu^{\prime \prime }$. The $\mu^{\prime }$ value curves of the three filling ratios decreased in the frequency test range; the variation trends of the $\mu^{\prime }$ of the three filling ratios were similar. In the range of 2–18 GH, the $\mu^{\prime }$ value decreased with increasing filling ratio and increased with decreasing filling ratio; this was because, in Fe NWs@SiO_2_-filled paraffin composites, as the filling ratio increased the SiO_2_ content became higher, leading to a decrease in the magnetic properties of the composites; thus, the $\mu^{\prime }$ value became smaller. The variation of $\mu^{\prime }$ was accompanied by many disordered peaks, which may have been caused by the suppression of the eddy current loss by a one-dimensional core–shell structure. The $\mu^{\prime \prime }$ value curves for 10 wt% and 30 wt% were the same and the $\mu^{\prime \prime }$ value curves for 50 wt% were overall higher than those for 10 wt% and 30 wt%. The 50 wt% had the highest filling ratio and exhibited the strongest magnetic loss. The $\mu^{\prime \prime }$ value curves of the three packing ratios showed a decreasing state and the samples regarding the three packing ratios showed many similar rebound peaks in the frequency range. This change may have come from the natural resonance, exchange resonance, and eddy current loss mechanism.

**Figure 7:**
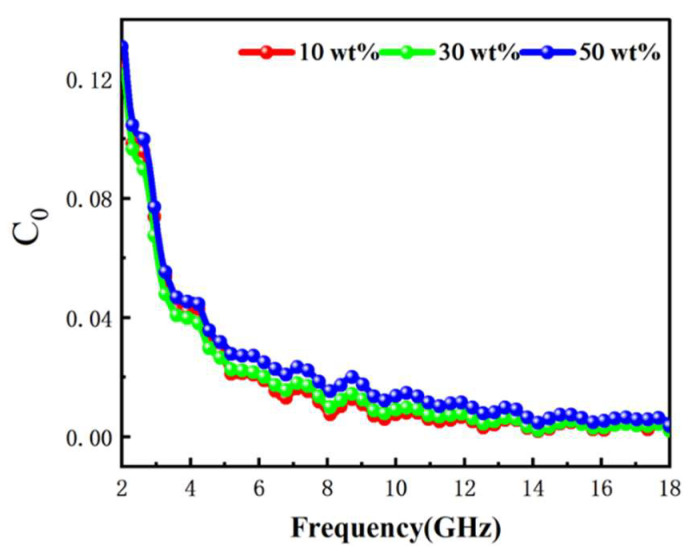
Eddy current loss (denoted by *C_0_*) of the Fe NWs@SiO_2_ composites with different filling ratios.

The magnetic losses in the microwave band are in the form of hysteresis, domain wall resonance, eddy current loss, natural resonance, and exchange resonance [51,52]. However, under weak external field conditions, hysteresis is usually neglected, while domain wall resonance occurs only in the 1–100 MHz range of multidomain magnetic materials [53,54]. Therefore, eddy current loss, natural resonance, and exchange resonance are the main influencing factors of magnetic loss. The eddy current loss concerning the thickness (d) and conductivity (σ) of the absorber can be expressed as the following equation [55]:

(4) $$\mu^{\prime \prime }=\frac{2\pi \mu_{0}{\left(\mu^{\prime }\right)}^{2}\sigma d^{2}f}{3}$$

(5) $$C_{0}=\mu^{\prime \prime }{\left(\mu^{\prime }\right)}^{-2}f^{-1}$$

where $\mu_{0}$ is the vacuum permeability. The $C_{0}$ values for different Fe NWs@SiO_2_ filling mass fractions are shown in Figure 7. When eddy current loss was the main magnetic loss mechanism, $C_{0}$ behaved as a constant and eddy current loss may have existed in the range of 12–18 GHz. The peak value at 4–10 GHz was mainly caused by natural resonance, while the fluctuation at high frequency (10–18 GHz) may have been caused by exchange resonance.

**Figure 8:**
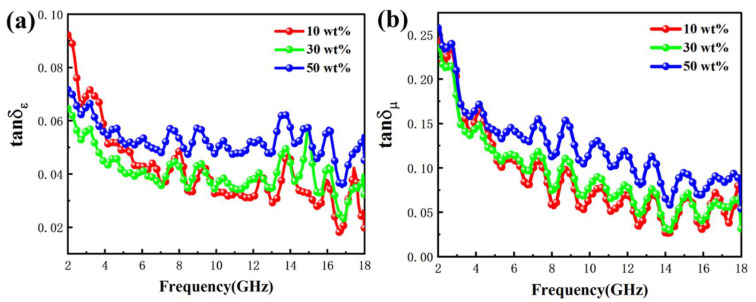
(**a**) Dielectric tangent loss and (**b**) magnetic tangent loss of the Fe NWs@SiO_2_ composites with different filling ratios.

To further analyze the dielectric and magnetic loss capabilities of the materials, the dielectric loss tangent curves ($\tan\delta_{\varepsilon }$) and magnetic loss tangent curves ($\tan\delta_{\mu }$) were calculated for different filling ratios, as shown in Figure 8a,b. It was obvious that the trends in $\tan\delta_{\varepsilon }$ and $\tan\delta_{\mu }$ curves were similar to the $\varepsilon^{\prime \prime }$ and $\mu^{\prime \prime }$ curves, indicating that both dielectric and magnetic losses contributed to the Fe NWs@SiO_2_ composites and both contributed to microwave absorption. For the whole frequency test range, the $\tan\delta_{\mu }$ values were significantly higher than the $\tan\delta_{\varepsilon }$ values, indicating high dielectric losses from magnetic losses. Therefore, the main absorption mechanism of microwave absorption in Fe NWs@SiO_2_ composites was magnetic loss.

Two aspects affect electromagnetic wave absorption performance. On the one hand, an electromagnetic wave enters the material as much as possible; on the other hand, the material can fully dissipate the incident electromagnetic wave energy [56]. The impedance coordination constant (Z) represents the ability of the electromagnetic wave to enter the material. If the Z value is closer to 1, it indicates that the electromagnetic wave enters the material more easily [57]. The attenuation constant (α) is used to describe the ability of the material to consume electromagnetic waves; the larger the value is, the stronger the ability to dissipate electromagnetic waves [58]. Only the most appropriate values of Z and α can make the material have the best absorbing properties. Z and α can be calculated by the following formula [59]:

(6) $$Z=\left|\frac{Z_{in}}{Z_{0}}\right|=|\sqrt{\frac{\mu_{r}}{\varepsilon_{r}}}\tanh\left[j\left(\frac{2\pi fd}{c}\right)\sqrt{\mu_{r}\varepsilon_{r}}\right]|$$

(7) $$\;\alpha =\frac{\sqrt{2}\pi f}{c}\times \sqrt{\left(\mu^{\prime \prime }\varepsilon^{\prime \prime }-\mu^{\prime }\varepsilon^{\prime }\right)+\sqrt{{\left(\mu^{\prime \prime }\varepsilon^{\prime \prime }-\mu^{\prime }\varepsilon^{\prime }\right)}^{2}+{\left(\mu^{\prime }\varepsilon^{\prime \prime }+\mu^{\prime \prime }\varepsilon^{\prime }\right)}^{2}}}$$

**Figure 9:**
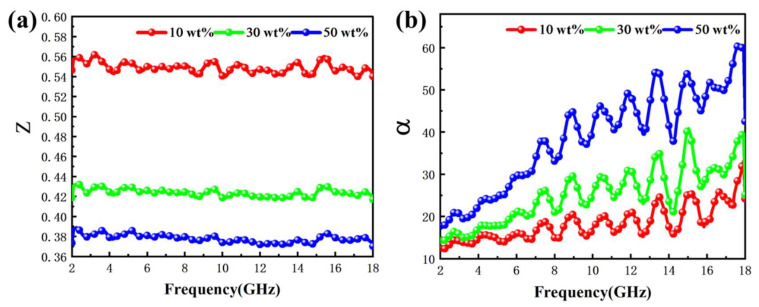
Frequency dependence of (**a**) impedance matching Z and (**b**) attenuation constant α for Fe NWs@SiO_2_ composites with different filling ratios.

The Z values of different Fe NWs@SiO_2_ composite filling mass ratios are shown in Figure 9. As can be seen from the figure, when the filling ratio increased, the Z value decreased as a whole, and the Z value of each filling ratio had a stable overall change trend, accompanied by slight fluctuations. When the filling ratio increased, the dielectric loss of Fe NWs@SiO_2_ increased and the charge storage capacity decreased, resulting in the overall decrease in the Z value. When an electromagnetic wave entered the material, a weak eddy current was generated inside the material, causing the peak value of Z. In the figure, the Z value decreased as the filler mass fraction increased, which was because the higher the filler mass fraction, the better the electrical conductivity. The α values for different filling mass ratios of Fe NWs@SiO_2_ composites are given in Figure 9b. The α values indicated the attenuation ability of Fe NWs@SiO_2_ composites to EMW; the larger the value, the greater the ability to consume EMW. It can be seen from the figure that the α value for the filling mass ratio of 50 wt% was greater than that of 10 wt% and 30 wt% in the whole frequency range, indicating better attenuation of the incident wave at 50 wt%. As the frequency increased, the α value also increased, indicating that the attenuation ability of the material increased with increasing frequency. The steep peaks that appear may have been related to the strong interfacial polarization. Thus, the filling mass ratio of 50 wt% had a poor impedance match, but the attenuation constant was the largest and EMW had the best absorption capacity, which corresponded to RL values for different filling mass fractions in Figure 4.

**Figure 10:**
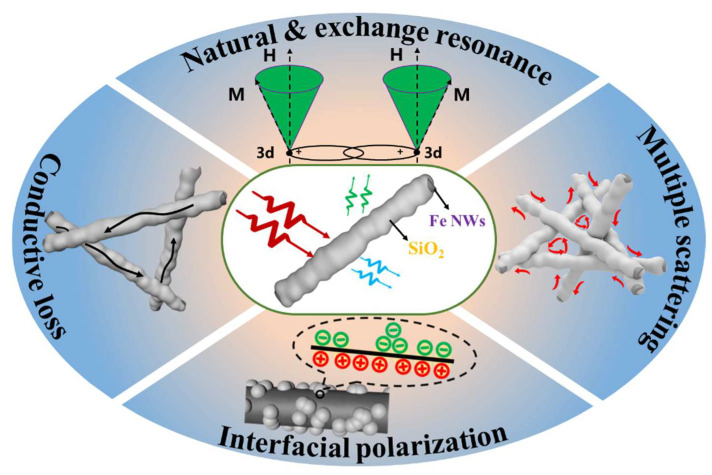
Wave absorption mechanism of Fe NWs@SiO_2_ composites.

The multiple wave-absorbing mechanisms of core–shell Fe NWs@SiO_2_ composites are shown in Figure 10. Firstly, the core–shell structured Fe NWs@SiO_2_ composites could generate abundant heterogeneous interfaces between the Fe NWs’ core, the SiO_2_ shell, and the paraffin. These heterogeneous interfaces would collect charges at the interface and at the junction to enhance the interfacial polarization and the dielectric loss would be increased when the material was under the alternating electromagnetic field [60]; secondly, the one-dimensional nanowire structure with a high aspect ratio formed a good network structure, which prolonged the microwave transmission path and facilitated microwave consumption. In addition, the nanowires intertwined with each other to cause multiple scattering of incident waves and further consumed the incident waves. Then, the natural resonance and exchange resonance generated by the one-dimensional Fe NWs increased the magnetic loss of the composite. In summary, these core–shell structured Fe NWs@SiO_2_ composites had good microwave consumption capability.

This study does have some shortcomings, such as a narrow effective absorption bandwidth of 2.88 GHz and a high-frequency absorption band, etc. The imaginary part of the dielectric constant became small after the FeNWs@SiO_2_ composite and the dielectric loss was not strong. To address these issues, we plan to introduce dielectric materials to promote polarization at heterogeneous interfaces, increase multi-interface polarization, improve dielectric loss, enhance absorption performance, and achieve broadband absorption. Additionally, we aim to improve the magnetic permeability and magnetic loss to absorb electromagnetic waves in the lower frequency band and gradually move towards multifunctional absorbing materials in future work.

## 3. Materials and Methods

### 3.1. Materials

All chemicals were of analytical grade and used without further purification. The Fe NWs were synthesized by a chemical reduction method under an external magnetic field according to our previous work [26]. Tetraethyl orthosilicate (TEOS) and aqueous ammonia (H_5_NO, 28%) were obtained from Aladdin Co., Ltd. (Shanghai, China). Anhydrous ethanol (C₂H₆O, 99.7%) was supplied by Kelong Co., Ltd. (Sichuan, China). All the solutions were prepared with deionized water (18.25 MΩ cm) and obtained from an ultrapure water system (GYJ2-20L-S) by Huachuang Co., Ltd. (Chongqing, China).

### 3.2. Synthesis

**Figure 11:**
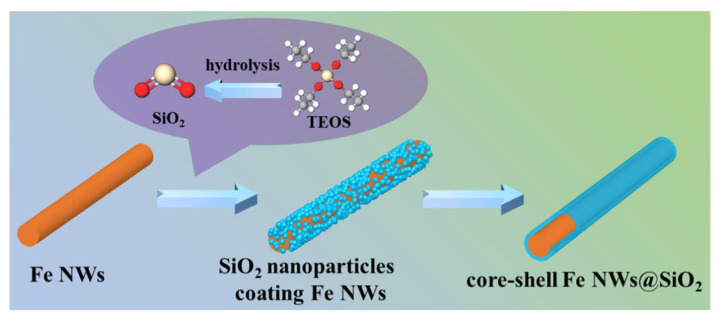
Schematic illustration of the synthesis of Fe NWs@SiO_2_.

Core–shell Fe NWs@SiO_2_ was synthesized by a simple liquid-phase hydrolysis method; the preparation process of core–shell Fe NWs@SiO_2_ is shown in Figure 11. The detailed steps can be seen below: firstly, configure the Fe NWs solution, add 0.5 g Fe NWs into the solution containing 150 mL anhydrous ethanol and 40 mL deionized water, and sonicate the solution for 10 min to disperse the Fe NWs uniformly; secondly, add 3 mL of ammonia with a mass fraction of 28% to the mixed solution obtained in step one and shake ultrasonically for 30 min to fully disperse; thirdly, add 4 mL TEOS dropwise into the mixed solution obtained from step two and maintain the reaction at 500 r/min at room temperature with mechanical stirring for 10 h until TEOS is fully hydrolyzed (TEOS hydrolysis equation as in Equation (8)); finally, obtain the Fe NWs@SiO_2_ core–shell composites by centrifugal washing and drying. FeNW@SiO_2_ and paraffin are mixed in a ratio of 1:9, 3:7, and 5:5 to prepare coaxial rings with an inner diameter of 3.04 mm, an outer diameter of 7 mm, and a thickness of 2 mm, respectively. Coaxial ring composites will be used to measure electromagnetic absorption performance.

(8) $${\left(C_{2}H_{5}O\right)}_{4}\mathrm{Si}+H_{2}O\to 4C_{2}H_{5}\mathrm{OH}+{\mathrm{SiO}}_{2}$$

### 3.3. Characterization

The morphology and structure of core–shell Fe NWs@SiO_2_ composites are characterized using scanning electron microscopy (FIB/SEM, ZEISS AURIGA, Oberkochen, Germany) and transmission electron microscopy (TEM, FEI F20, Thermo Scientific, Waltham, MA, USA), the elemental compositions using energy dispersive spectroscopy (EDS) attach to the SEM, the crystal structure and surface elemental composition are determined using X-ray powder diffraction (XRD, Rigaku Ultima IV, Tokyo, Japan) and energy dispersive X-ray spectroscopy (EDS), magnetic properties are measured by vibrating sample magnetometer (VSM, Lakeshore 7404, Columbus, OH, USA), thermal stability is measured by thermogravimetric analysis (TGA, Mettler Toledo TGA/DSC3+, Shanghai, China), and the electromagnetic parameters are measured by a vector network analyzer (Agilent N5234A, Santa Clara, CA, USA) in the frequency range of 2–18 GHz.

## 4. Conclusions

In this study, we successfully coated SiO_2_ on the surface of Fe NWs using a simple liquid-phase hydrolysis method, resulting in the production of Fe NWs@SiO_2_ composites with a core–shell structure. The results showed that the Fe NWs had significantly enhanced antioxidant properties and that the sample filled with 50 wt% had the best composite performance. At the matching thickness of 7.25 mm, the *RL_min_* could reach −54.88 dB at 13.52 GHz and the EAB could reach 2.88 GHz in the range of 8.96–17.12 GHz. The improvement in the microwave absorption performance of Fe NWs@SiO_2_ composites with core–shell structure could be attributed to the enrichment of interfacial polarization, the reduction of dielectric constant, and the optimization of impedance matching. In addition, the one-dimensional nanowire structure and complex network structure facilitated the transmission and scattering of incident waves and enhanced the microwave absorption properties. This study provided a simple and effective process for the production of Fe NWs@SiO_2_ composites with core–shell structures with high absorption and oxidation resistance for future practical applications. In future research, the focus will be on multifunctional wave-absorbing materials and the broadband absorption of electromagnetic waves, which are important for the construction of broadband stealth weapon platforms.

## Figures and Tables

**Figure A2:**
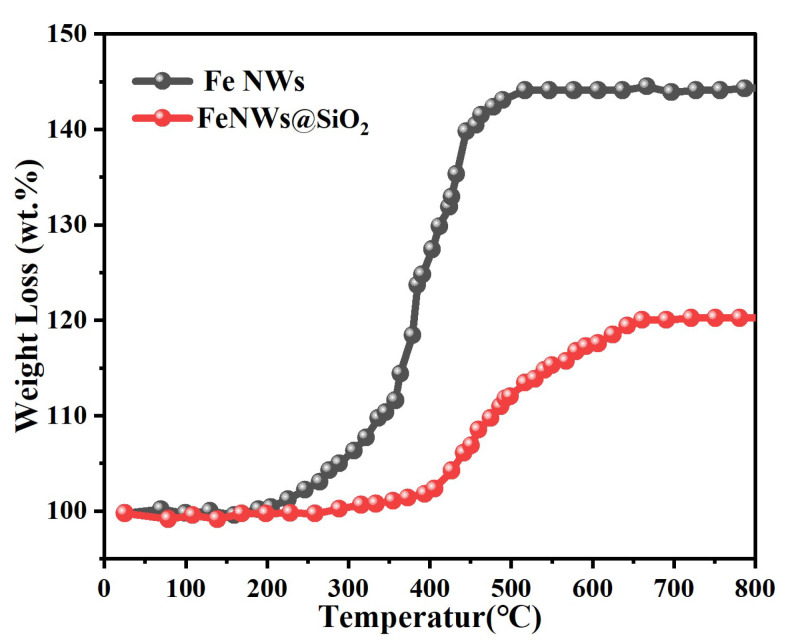
TGA curves of Fe NWs and core–shell Fe NWs@SiO_2_ in air.

## Data Availability

Not applicable.

## Appendix A

Experimental evidence: Thermo-gravimetric analysis of Fe NWs and Fe NWs@SiO_2_ was performed. As shown in Figure A2, untreated Fe NWs began to oxidize at 159 °C and showed a mass increase in an air atmosphere and oxidized completely at 500 °C. The mass of SiO_2_-coated Fe NWs increased only at 400 °C and became stable at 650 °C. At 800 °C, the mass fraction of Fe NWs and Fe NWs@SiO_2_ was 144.31% and 120.1%, respectively, which was consistent with the mass increase introduced by the oxidation of iron oxide (Fe_2_O_3_). In addition, the oxidation temperature of Fe NWs@SiO_2_ was 400 °C, compared with 159 °C for rapid oxidation of Fe NWs. In summary, Fe NWs@SiO_2_ had good thermal stability.
